# Oral microbiome dysbiosis is associated with chronic respiratory diseases: evidence from a population-based study and a hospital cohort

**DOI:** 10.3389/fpubh.2025.1696041

**Published:** 2025-10-30

**Authors:** Baolin Jia, Xiaojuan Wu, Gaoyan He, Qiang Wang, Li Guan, Jun Ren, Guixin Li, Xianjie Zheng, Sen Yang

**Affiliations:** ^1^Department of Oral and Maxillofacial Surgery, Suining Central Hospital, Suining, Sichuan, China; ^2^Department of Respiratory Medicine and Critical Care Medicine, Suining Central Hospital, Suining, Sichuan, China

**Keywords:** oral microbiome, chronic respiratory disease, alpha diversity, beta diversity, NHANES, linear discriminant analysis effect size (LEfSe), 16s ribosomal RNA (rRNA) sequencing

## Abstract

**Background:**

The oral microbiome has been increasingly recognized for its role in systemic health through the oral–lung axis. However, population-level evidence linking oral microbial diversity and composition with chronic respiratory diseases (CRD) remains limited.

**Methods:**

We analyzed data from 4,384 adults in the 2009–2012 National Health and Nutrition Examination Survey (NHANES), defining CRD by self-reported chronic obstructive pulmonary disease (COPD), asthma, emphysema, or chronic bronchitis. Oral rinse samples underwent 16S ribosomal RNA (16S rRNA) V1–V3 sequencing. Alpha diversity, including observed amplicon sequence variants (ASVs), Faith’s phylogenetic diversity (Faith’s PD), Shannon–Weiner index, and Simpson index, and beta diversity, including Bray–Curtis, weighted UniFrac, and unweighted UniFrac distances, were assessed. Associations with CRD were examined using weighted logistic regression and restricted cubic splines (RCS). Differential genus abundance was identified by Wilcoxon tests with false discovery rate correction. A random forest model integrated microbial and clinical features. An independent hospital cohort was additionally profiled by 16S rRNA sequencing, and genus-level differences were assessed with linear discriminant analysis effect size (LEfSe) to validate NHANES findings.

**Results:**

Higher alpha diversity was inversely associated with CRD risk; each standard deviation increase in observed ASVs and Faith’s PD reduced CRD odds by 19 and 17%, respectively (*p* < 0.05). Beta diversity showed significant community-level separation by CRD status (*p* = 0.01). Several genera, including *Rothia* and *Veillonella*, were enriched in CRD, whereas *Prevotella*, *Haemophilus*, and *Neisseria* were more abundant in non-CRD individuals. The random forest model achieved an area under the curve (AUC) of 0.65. In the hospital cohort, compositional shifts were consistent with NHANES findings, and LEfSe confirmed the depletion of *Alloprevotella* and *Peptostreptococcus* in CRD patients.

**Conclusion:**

Oral microbial diversity and composition were significantly associated with CRD across both a representative U. S. population and a hospital cohort. Select genera and diversity indices may serve as non-invasive biomarkers for respiratory health, warranting further validation in longitudinal and mechanistic studies.

## 1 Introduction

The oral cavity harbors a complex ecosystem of microorganisms—including bacteria, fungi, protozoa, mycoplasmas, and viruses—alongside teeth, gingiva, tongue, mucosa, and saliva (1). These microbes often form biofilms that support immune regulation, epithelial defense, and microbial homeostasis (2–4).

When this balance is disrupted, pathogenic species may cross local barriers and spread via the airway, bloodstream, or digestive tract, influencing systemic health (5). Oral pathogens have been implicated in cardiovascular disease, diabetes, endocarditis, atherosclerosis, rheumatoid arthritis, and cancers (6–10). In recent years, the concept of an “oral–lung axis” has drawn attention. Oral microorganisms can reach the lower respiratory tract through aspiration or mucosal migration and may contribute to chronic respiratory diseases (CRD), such as chronic obstructive pulmonary disease (COPD) and asthma (11). Indeed, genera such as *Veillonella, Prevotella,* and *Rothia*—common in the oral cavity—are frequently enriched in sputum and bronchoalveolar lavage fluid of COPD patients (12).

CRD, including COPD, asthma, chronic bronchitis, and emphysema, are major causes of morbidity and mortality worldwide, with disproportionate impact in low- and middle-income countries (13, 14). Known risk factors such as smoking, air pollution, and occupational exposures play important roles, but their predictive value for early detection remains limited (15). Novel biomarkers, particularly those linked to the oral microbiome, may enhance CRD risk assessment and prevention. However, despite growing evidence, population-based studies directly linking oral microbial diversity to aggregated CRD outcomes remain scarce.

Previous studies have suggested enrichment of oral bacteria in lung samples from CRD patients. However, most focused on single diseases, yielding inconsistent findings, while in clinical practice these conditions frequently overlap. Our study uniquely addresses this gap by aggregating COPD, asthma, chronic bronchitis, and emphysema into a unified CRD outcome, thereby reflecting real-world comorbidity and improving statistical power. To our knowledge, this is the first study to integrate a nationally representative cohort (NHANES) with a hospital-based cohort to investigate the association between oral microbial diversity and aggregated CRD outcomes. This two-stage design strengthens epidemiologic evidence and highlights potential microbial biomarkers with implications for both public health and clinical care.

## 2 Materials and methods

### 2.1 NHANES study

**Figure 1:**
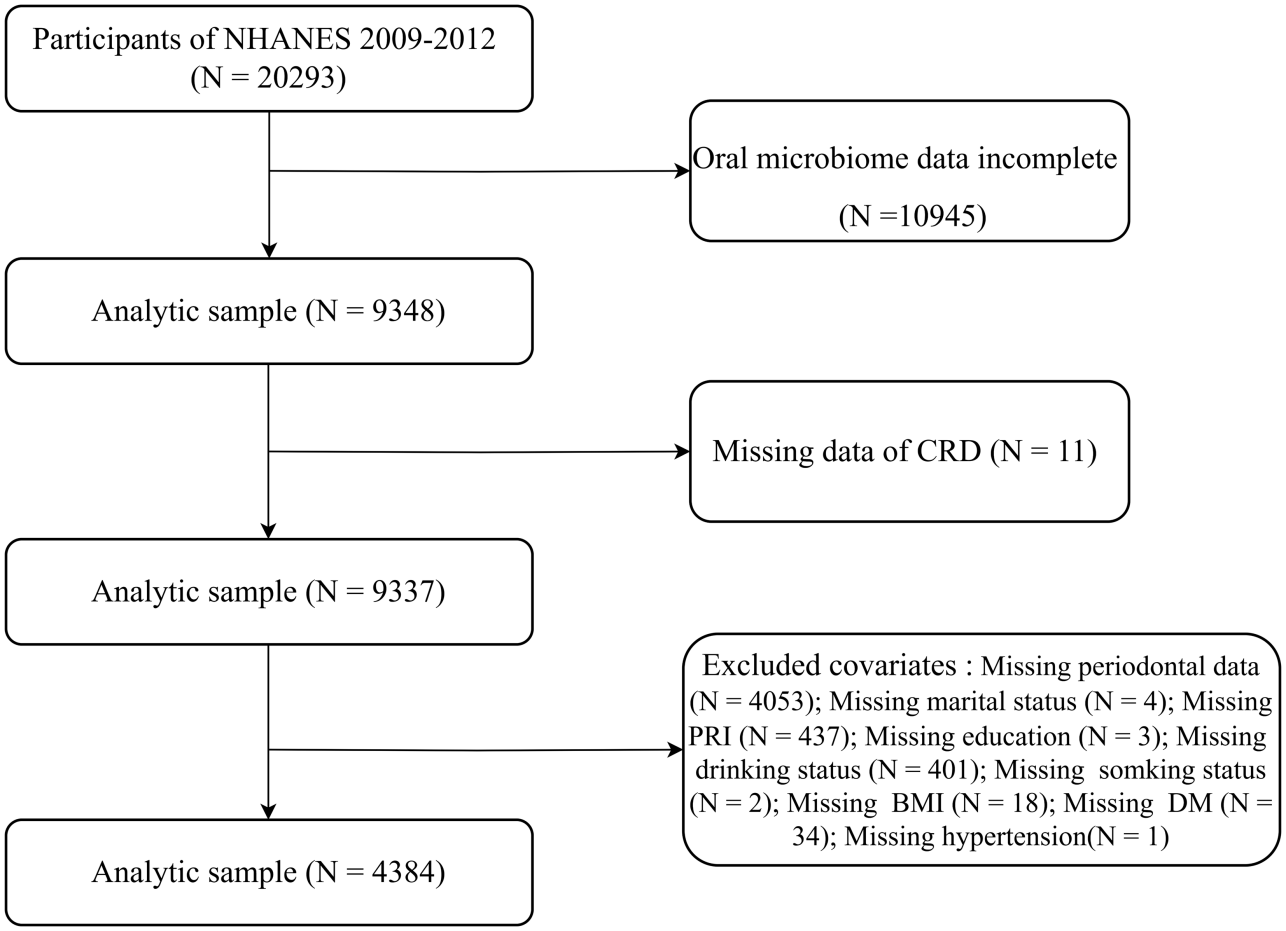
Flowchart depicting participant inclusion and exclusion criteria for the study in the NHANES.

NHANES is a continuous, nationally representative cross-sectional study that uses a stratified, multistage probability sampling method to assess the health of the non-institutionalized U. S. population (16). The study protocol was approved by the National Center for Health Statistics Ethics Review Board, and all participants provided written informed consent. This study followed the STROBE reporting guidelines (17). For the present analysis, we combined data from the 2009–2010 and 2011–2012 NHANES cycles. Among 20,293 participants initially enrolled, those without oral microbiome sequencing data (*n* = 10,945), missing CRD status (*n* = 11), or incomplete covariate information (*n* = 4,953) were excluded. Missing covariate data included periodontal measures (*n* = 4,053), poverty income ratio (*n* = 437), alcohol use (*n* = 401), diabetes status (*n* = 34), body mass index (BMI) (*n* = 18), marital status (*n* = 4), smoking status (*n* = 2), education level (*n* = 3), and hypertension diagnosis (*n* = 1). A total of 4,384 participants were included in the final analytic sample (Figure 1).

Oral rinse samples collected during the 2009–2010 and 2011–2012 cycles were used for oral microbiome profiling. Genomic DNA was extracted and amplified targeting the V1–V3 regions of the 16S ribosomal RNA (rRNA) gene, followed by high-throughput sequencing. Raw sequence reads underwent standard quality control procedures to generate amplicon sequence variants (ASVs), with taxonomic classification assigned from phylum to genus levels using curated reference databases. Alpha diversity was assessed using Observed ASVs, Faith’s phylogenetic diversity (Faith’s PD), Shannon–Weiner index, and Simpson index, calculated from rarefied datasets normalized to 10,000 reads per sample. Each metric was averaged across 10 independent subsamplings to enhance stability (18). Beta diversity was evaluated using Bray–Curtis dissimilarity (19), unweighted UniFrac, and weighted UniFrac distances to capture between-sample variation.

The CRD were defined as having at least one of the following conditions: COPD, asthma, emphysema, or chronic bronchitis. COPD was identified based on either a self-reported medical diagnosis or a post-bronchodilator FEV₁/FVC ratio <0.70 from spirometry testing. Asthma, emphysema, and chronic bronchitis were defined using participants’ responses to structured questionnaire items indicating whether a healthcare professional had ever diagnosed them with these conditions. Covariates included both continuous (age, BMI) and categorical variables (sex, race/ethnicity, education level, marital status, and poverty income ratio [<1.3, 1.3–3.5, ≥3.5]). Smoking status was categorized as never, former, or current; alcohol use was classified as never, former, mild, moderate, or heavy; and physical activity was divided into low (<500 MET-min/week) and high (≥500 MET-min/week), according to NHANES recommendations (20). Hypertension, diabetes, and hyperlipidemia were defined based on a combination of self-reported diagnoses, physical examination findings, and laboratory results (Supplementary Table S1). Oral health–related covariates included periodontitis severity, categorized using CDC/AAP criteria based on clinical attachment loss (CAL) and probing depth (PD) (21), as well as oral hygiene practices such as dental floss and mouthwash use in the past 7 days (yes/no).

### 2.2 Clinical research

Consecutive adult outpatients were recruited from the Department of Respiratory Medicine at Suining Central Hospital between August and September 2025. Eligible participants were aged 30 years or older and provided written informed consent. Participants were divided into two groups: the CRD group (*n* = 49), comprising patients with a clinical diagnosis of COPD, asthma, chronic bronchitis, or emphysema; and the control group (*n* = 46), consisting of outpatients without a history of chronic respiratory diseases. The study was approved by the Ethics Committee of Suining Central Hospital (approval number KYLLKS20250140). Exclusion criteria were: (1) use of antibiotics within the past month; (2) acute oral disease at the time of recruitment; and (3) presence of severe systemic illnesses.

The following data were recorded: (1) Demographic data: age, sex, occupation, height, weight, and BMI; (2) Lifestyle factors: smoking and drinking history, oral hygiene behaviors (e.g., use of dental floss and mouthwash); (3) Medical history: comorbidities and medication use; (4) Clinical examination: pulmonary function testing, disease diagnosis, and periodontal status. Saliva samples (10–15 mL) were collected from each participant and immediately stored at −80 °C. Microbial DNA was extracted, and the V3–V4 regions of the bacterial 16S rRNA gene were amplified and subjected to high-throughput sequencing. Sequencing data were processed using standard quality control pipelines to generate microbial taxonomic profiles and diversity measures.

### 2.3 Statistical analysis

All analyses were conducted using R software (version 4.4.3).

For the NHANES data, complex survey design weights were incorporated following analytic guidelines. A two-sided *p*-value < 0.05 was considered statistically significant. Continuous variables were summarized as means ± standard deviations (SD) and compared between CRD and non-CRD groups using weighted t-tests. Categorical variables were reported as frequencies with weighted percentages and compared using the Rao–Scott chi-square test. Weighted logistic regression was applied to examine associations between alpha diversity indices and prevalence of CRD, with results expressed as odds ratios (ORs) and 95% confidence intervals (CIs) per SD increase. Tertile-based analyses and tests for trend were also conducted. Restricted cubic spline (RCS) models were used to evaluate dose–response relationships and potential nonlinearity. Subgroup analyses were performed for Observed ASVs and Faith’s PD across age, sex, race/ethnicity, BMI, and smoking status, with interaction terms to assess effect modification. Beta diversity was evaluated using principal coordinates analysis (PCoA) and PERMANOVA based on Bray–Curtis, unweighted UniFrac, and weighted UniFrac distances. At the genus level, genera with <5% prevalence were excluded, and differentially abundant genera were identified using Wilcoxon tests with false discovery rate (FDR) correction. Key results were visualized with heatmaps and boxplots. A random forest model was constructed using the top 10 differentially abundant genera, alpha diversity indices, and selected clinical variables to classify CRD status, with performance assessed by receiver operating characteristic (ROC) curves and area under the curve (AUC). Sensitivity analyses excluded participants who had recently used antibiotics, and an additional analysis was performed incorporating HEI-2015 (diet quality) as a covariate in the regression models.

For the hospital cohort, baseline characteristics between CRD and non-CRD participants were compared using the independent sample *t*-test for continuous variables and the chi-square test for categorical variables. α-diversity indices (Observed species, Chao1, Shannon, and Simpson) were calculated in R (v4.4.3) based on rarefied ASV tables. Group differences in α-diversity were evaluated using the Wilcoxon rank-sum test. β-diversity was assessed using R (v4.4.3) based on Bray–Curtis and Jaccard distance metrics. Principal coordinates analysis (PCoA) was performed to visualize community dissimilarities, and PERMANOVA (999 permutations) was applied to test for significance between CRD and non-CRD groups. At the genus level, taxa with a prevalence <5% across samples were excluded from analysis. Relative abundances were compared between groups using the Wilcoxon rank-sum test, with false discovery rate (FDR) correction applied for multiple testing. Differentially abundant taxa were further identified using linear discriminant analysis effect size (LEfSe) with an LDA score threshold of 2.0. Taxonomic cladograms were generated to illustrate taxa enriched in CRD or non-CRD participants. Microbial co-occurrence networks were constructed at multiple taxonomic levels using SparCC correlation analysis, and network modules were visualized to explore potential ecological interactions.

## 3 Results

### 3.1 Baseline characteristics

In the NHANES analysis, among an estimated 93,587,279 U. S. adults, the prevalence of CRD was 19.7% (Table 1). Compared with those without CRD, affected individuals were slightly older (49.12 ± 11.03 vs. 47.56 ± 10.61), more likely to be non-Hispanic White (73.89% vs. 67.56%), and had higher rates of divorce, widowhood, or separation (22.89% vs. 17.42%). They also showed greater obesity prevalence (BMI ≥ 30: 43.24% vs. 36.63%), lower income (PIR < 1.3: 20.43% vs. 17.71%), and higher smoking rates (29.39% vs. 16.70%). The CRD group additionally exhibited a higher prevalence of moderate-to-severe periodontitis (35.26% vs. 30.08%), hypertension (43.23% vs. 34.41%), and hyperlipidemia (78.44% vs. 72.96%). No significant differences were found in sex, education, diabetes, physical activity, or oral hygiene behaviors (all *p* > 0.05). Importantly, participants with CRD had significantly lower oral microbial diversity across Observed ASVs, Faith’s phylogenetic diversity, and the Shannon index (all *p* < 0.05).

**Table 1:** Baseline characteristics of participants by CRD status.

Characteristic	Overall *N* = 93,587,279^1^	Without CRD *N* = 75,154,453^1^	With CRD *N* = 18,432,826^1^	*P*-value^2^
Age (years)	47.87 ± 10.71	47.56 ± 10.61	49.12 ± 11.03	0.004
Observed ASVs	126.14 ± 40.25	127.58 ± 40.94	120.28 ± 36.71	<0.001
Faith’s phylogenetic diversity	14.21 ± 3.19	14.31 ± 3.22	13.81 ± 3.02	<0.001
Shannon-Weiner index	4.59 ± 0.65	4.61 ± 0.66	4.53 ± 0.62	0.027
Simpson index	0.90 ± 0.06	0.90 ± 0.06	0.90 ± 0.06	0.785
Sex				0.103
Male	2,254 (51.38%)	1,867 (52.27%)	387 (47.75%)	
Female	2,130 (48.62%)	1,690 (47.73%)	440 (52.25%)	
Race/ethnicity				<0.001
Non-Hispanic White	1,775 (68.81%)	1,374 (67.56%)	401 (73.89%)	
Non-Hispanic Black	998 (11.11%)	785 (10.87%)	213 (12.07%)	
Mexican American	710 (8.22%)	642 (9.38%)	68 (3.49%)	
Other Hispanic	448 (5.23%)	366 (5.39%)	82 (4.57%)	
Other Race	453 (6.64%)	390 (6.80%)	63 (5.98%)	
Marital status				<0.001
Married/Living with Partner	2,880 (70.33%)	2,397 (71.74%)	483 (64.59%)	
Widowed/Divorced/Separated	954 (18.50%)	727 (17.42%)	227 (22.89%)	
Never married	550 (11.17%)	433 (10.84%)	117 (12.52%)	
BMI				0.013
<25	1,103 (26.38%)	900 (26.69%)	203 (25.11%)	
25–29.9	1,508 (35.69%)	1,264 (36.68%)	244 (31.65%)	
≥30	1,773 (37.93%)	1,393 (36.63%)	380 (43.24%)	
Education				0.355
Below high school	407 (4.64%)	353 (4.90%)	54 (3.60%)	
High school	1,552 (30.38%)	1,248 (30.28%)	304 (30.79%)	
Above high school	2,425 (64.98%)	1,956 (64.82%)	469 (65.62%)	
PIR				0.012
<1.3	1,327 (18.24%)	1,027 (17.71%)	300 (20.43%)	
1.3–3.5	1,501 (32.71%)	1,224 (31.86%)	277 (36.21%)	
>3.5	1,556 (49.04%)	1,306 (50.44%)	250 (43.36%)	
Smoking status				<0.001
Never	2,427 (55.58%)	2,069 (58.47%)	358 (43.81%)	
Former	1,013 (25.22%)	812 (24.83%)	201 (26.80%)	
Now	944 (19.20%)	676 (16.70%)	268 (29.39%)	
Alcohol intake				0.053
Never	498 (8.38%)	428 (8.98%)	70 (5.97%)	
Former	733 (14.15%)	589 (13.79%)	144 (15.63%)	
Mild	1,501 (38.47%)	1,220 (38.98%)	281 (36.39%)	
Moderate	716 (18.81%)	578 (18.55%)	138 (19.86%)	
Heavy	936 (20.18%)	742 (19.70%)	194 (22.15%)	
Physical activity				0.257
Low	741 (16.53%)	590 (16.17%)	151 (18.02%)	
High	3,643 (83.47%)	2,967 (83.83%)	676 (81.98%)	
Flossing behavior				0.365
No	1,427 (28.60%)	1,152 (29.02%)	275 (26.92%)	
Yes	2,957 (71.40%)	2,405 (70.98%)	552 (73.08%)	
Mouthwash behavior				0.484
No	1,869 (46.59%)	1,517 (46.24%)	352 (48.05%)	
Yes	2,515 (53.41%)	2,040 (53.76%)	475 (51.95%)	
Periodontitis				0.009
No/Mild periodontitis	2,710 (68.90%)	2,201 (69.92%)	509 (64.74%)	
Moderate/Severe periodontitis	1,674 (31.10%)	1,356 (30.08%)	318 (35.26%)	
Hypertension				<0.001
No	2,637 (63.85%)	2,211 (65.59%)	426 (56.77%)	
Yes	1,747 (36.15%)	1,346 (34.41%)	401 (43.23%)	
Diabetes				0.051
No	3,620 (87.45%)	2,967 (88.12%)	653 (84.73%)	
Yes	764 (12.55%)	590 (11.88%)	174 (15.27%)	
Hyperlipidemia				0.026
No	1,190 (25.96%)	986 (27.04%)	204 (21.56%)	
Yes	3,194 (74.04%)	2,571 (72.96%)	623 (78.44%)	

^1^*n* (unweighted) (%).

^2^Design-based *t*-test; Pearson’s X^2: Rao and Scott adjustment.

In the hospital-based cohort, 95 participants were enrolled, including 49 with CRD and 46 without CRD (Table 2). Patients with CRD were older (62.94 ± 9.58 vs. 50.63 ± 12.11), more likely to be current smokers (46.94% vs. 21.74%), and more frequently lived in rural areas (73.47% vs. 47.83%). They also had a higher prevalence of periodontitis (48.98% vs. 17.39%), whereas no significant group differences were observed for BMI, sex, alcohol use, marital status, education, diabetes, hypertension, hyperlipidemia, flossing, or mouthwash behaviors (all *p* > 0.05). Similar to NHANES, the CRD group demonstrated significantly lower alpha diversity indices, including Observed species richness, Chao1, Shannon, and Simpson indices (all *p* < 0.001).

**Table 2:** Associations between oral microbial alpha diversity indices and CRD in unadjusted and fully adjusted logistic regression models.

CRD	Model 1		Model 2		Model 3	
	OR (95% CI)	*P*-value	OR (95% CI)	*P*-value	OR (95% CI)	*P*-value
Observed ASVs						
Per SD increase	0.82 (0.74, 0.90)	<0.001	0.84 (0.75, 0.94)	0.004	0.81 (0.70, 0.93)	0.009
Q1	Ref		Ref		Ref	
Q2	0.77 (0.59, 1.02)	0.064	0.81 (0.60,1.07)	0.133	0.81 (0.57, 1.16)	0.205
Q3	0.67 (0.52, 0.87)	0.003	0.71 (0.53, 0.95)	0.021	0.65 (0.46, 0.92)	0.023
*P* for trend		0.003		0.021		0.022
Faith’s phylogenetic diversity						
Per SD increase	0.84 (0.77, 0.93)	<0.001	0.86 (0.77, 0.97)	0.016	0.83 (0.71, 0.96)	0.019
Q1	Ref		Ref		Ref	
Q2	0.81 (0.64, 1.03)	0.086	0.83 (0.64, 1.07)	0.135	0.80 (0.57, 1.11)	0.141
Q3	0.70 (0.55, 0.88)	0.004	0.73 (0.55, 0.98)	0.033	0.65 (0.46, 0.93)	0.025
*P* for trend		0.029		0.027		0.020
Shannon-Weiner index						
Per SD increase	0.89 (0.81, 0.99)	0.026	0.91 (0.82, 1.01)	0.082	0.89 (0.79, 1.01)	0.071
Q1	Ref		Ref		Ref	
Q2	0.87 (0.67, 1.14)	0.307	0.91 (0.69, 1.20)	0.487	0.93 (0.66, 1.31)	0.633
Q3	0.72 (0.55, 0.95)	0.021	0.75 (0.57, 1.01)	0.061	0.72 (0.50, 1.03)	0.069
*P* for trend		0.018		0.054		0.060
Simpson index						
Per SD increase	0.99 (0.90, 1.08)	0.781	0.99 (0.90, 1.09)	0.838	0.97 (0.88, 1.08)	0.574
Q1	Ref		Ref		Ref	
Q2	1.04 (0.77, 1.41)	0.791	1.05 (0.75, 1.45)	0.781	1.01 (0.69, 1.49)	0.886
Q3	0.79 (0.60, 1.04)	0.093	0.78 (0.59, 1.05)	0.099	0.76 (0.53, 1.07)	0.095
*P* for trend		0.089		0.096		0.086

Results are presented as odds ratios (ORs) with 95% confidence intervals (CIs).

Model 1 is unadjusted.

Model 2 is adjusted for age, sex, race/ethnicity, marital status, PIR, education and BMI.

Model 3 is further adjusted for smoking status, physical activity, alcohol intake, Flossing behavior, mouthwash behavior, hypertension, DM, hyperlipidemia and periodontitis.

### 3.2 Association of oral microbial alpha diversity with CRD

**Figure 2:**
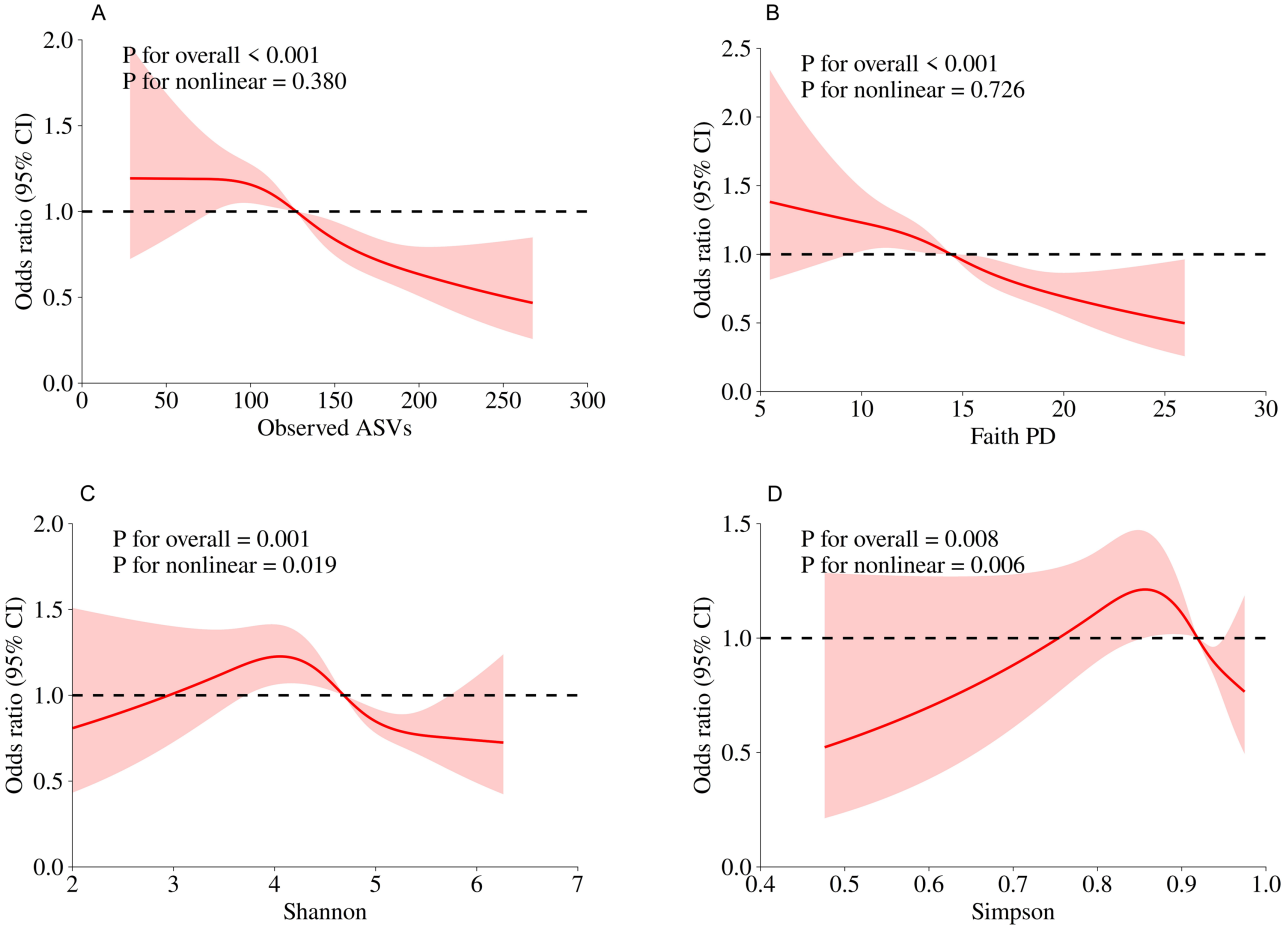
Dose–response relationships between oral microbial alpha diversity indices and CRD risk assessed by restricted cubic spline models in the NHANES. **(A)** Observed ASVs; **(B)** Faith’s Phylogenetic Diversity; **(C)** Shannon index; **(D)** Simpson index.

**Figure 3:**
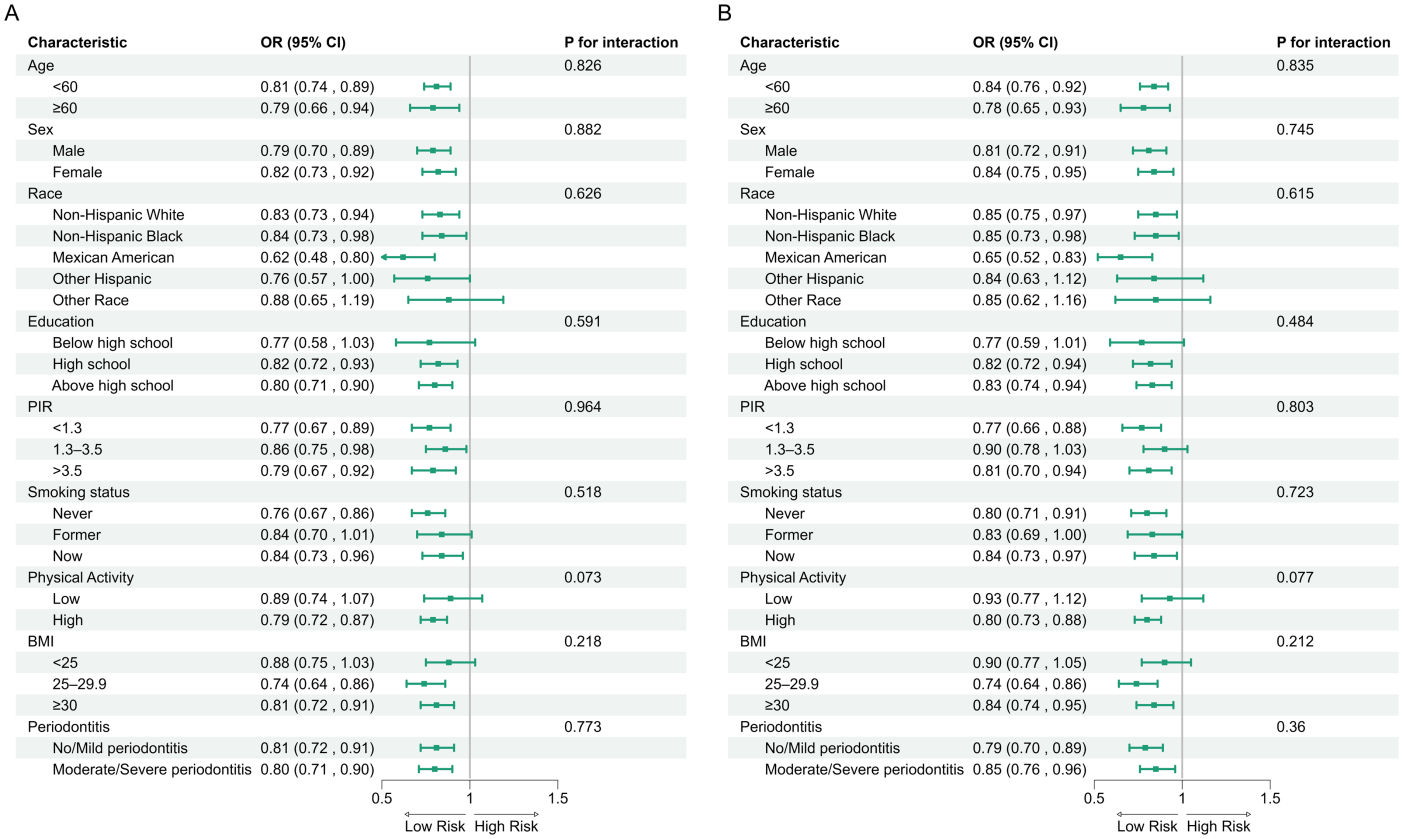
Subgroup analyses of associations between alpha diversity indices and CRD risk in the NHANES. **(A)** Observed ASVs; **(B)** Faith’s phylogenetic diversity.

In the NHANES cohort (Table 3), individuals with higher Observed ASVs had lower odds of CRD. Specifically, each SD increase in Observed ASVs was associated with OR = 0.82 (95% CI: 0.74–0.90, *p* < 0.001) in the unadjusted model and OR = 0.81 (95% CI: 0.70–0.93, *p* = 0.009) after adjusting for covariates. Participants in the highest tertile (Q3) had OR = 0.65 (95% CI: 0.46–0.92, *p* = 0.023) compared with the lowest tertile (Q1), with a significant trend across tertiles (*p* for trend = 0.022). Faith’s Phylogenetic Diversity showed similar associations (fully adjusted OR per SD = 0.83, 95% CI: 0.71–0.96, *p* = 0.019; Q3 vs. Q1 OR = 0.65, 95% CI: 0.46–0.93, *p* = 0.025, *p* for trend = 0.020). For the Shannon–Weiner index, the fully adjusted OR per SD was 0.89 (95% CI: 0.79–1.01, *p* = 0.071), and no significant association was observed for the Simpson index (OR per SD = 0.97, 95% CI: 0.88–1.08, *p* = 0.574). Dose–response curves using restricted cubic splines confirmed linear inverse associations for Observed ASVs and Faith’s PD, while Shannon and Simpson indices exhibited non-linear patterns (Figures 2A–D). Subgroup analyses showed consistent inverse associations across age, sex, race/ethnicity, education, smoking status, physical activity, BMI, and periodontitis severity (Figures 3A,B).

**Table 3:** Baseline characteristics of participants in the hospital-based cohort according to CRD status.

Variables	Total (*n* = 95)	Without CRD (*n* = 46)	Without CRD (*n* = 49)	*P-*value
Age, mean ± SD	56.98 ± 12.46	50.63 ± 12.11	62.94 ± 9.58	<0.001
BMI, mean ± SD	23.77 ± 4.10	24.03 ± 3.79	23.53 ± 4.39	0.554
Observed, mean ± SD	137.97 ± 47.96	159.74 ± 41.46	117.53 ± 44.86	<0.001
Chao1, mean ± SD	140.00 ± 49.25	162.16 ± 42.50	119.19 ± 46.31	<0.001
Shannon, mean ± SD	3.61 ± 0.58	3.86 ± 0.49	3.37 ± 0.57	<0.001
Simpson, mean ± SD	0.93 ± 0.05	0.95 ± 0.03	0.91 ± 0.06	<0.001
Sex, *n* (%)				0.130
Female	34 (35.79)	20 (43.48)	14 (28.57)	
Male	61 (64.21)	26 (56.52)	35 (71.43)	
Smoking, *n* (%)				0.010
No	62 (65.26)	36 (78.26)	26 (53.06)	
Yes	33 (34.74)	10 (21.74)	23 (46.94)	
Drinking, *n* (%)				0.658
No	64 (67.37)	32 (69.57)	32 (65.31)	
Yes	31 (32.63)	14 (30.43)	17 (34.69)	
Marital status, *n* (%)				0.052
No	8 (8.42)	7 (15.22)	1 (2.04)	
Yes	87 (91.58)	39 (84.78)	48 (97.96)	
Education, *n* (%)				0.232
High school or lower	78 (82.11)	40 (86.96)	38 (77.55)	
Above high school	17 (17.89)	6 (13.04)	11 (22.45)	
Residence, *n* (%)				0.010
Rural	58 (61.05)	22 (47.83)	36 (73.47)	
Urban	37 (38.95)	24 (52.17)	13 (26.53)	
DM, *n* (%)				0.138
No	88 (92.63)	45 (97.83)	43 (87.76)	
Yes	7 (7.37)	1 (2.17)	6 (12.24)	
Hypertension, *n* (%)				0.082
No	83 (87.37)	43 (93.48)	40 (81.63)	
Yes	12 (12.63)	3 (6.52)	9 (18.37)	
Hyperlipidemia, *n* (%)				0.259
No	76 (80.00)	39 (84.78)	37 (75.51)	
Yes	19 (20.00)	7 (15.22)	12 (24.49)	
Flossing behavior, *n* (%)				0.948
No	72 (75.79)	35 (76.09)	37 (75.51)	
Yes	23 (24.21)	11 (23.91)	12 (24.49)	
Mouthwash behavior, *n* (%)				0.973
No	68 (71.58)	33 (71.74)	35 (71.43)	
Yes	27 (28.42)	13 (28.26)	14 (28.57)	
Periodontitis, *n* (%)				0.001
No	63 (66.32)	38 (82.61)	25 (51.02)	
Yes	32 (33.68)	8 (17.39)	24 (48.98)	

Continuous variables are presented as mean ± standard deviation (SD) and were compared using independent-sample *t*-tests. Categorical variables are presented as counts and percentages (*n*, %) and were compared using chi-square tests. CRD was defined as a clinical diagnosis of COPD, asthma, chronic bronchitis, or emphysema. BMI, body mass index; DM, diabetes mellitus. *p*-values indicate the statistical significance of differences between participants with and without CRD.

**Figure 4:**
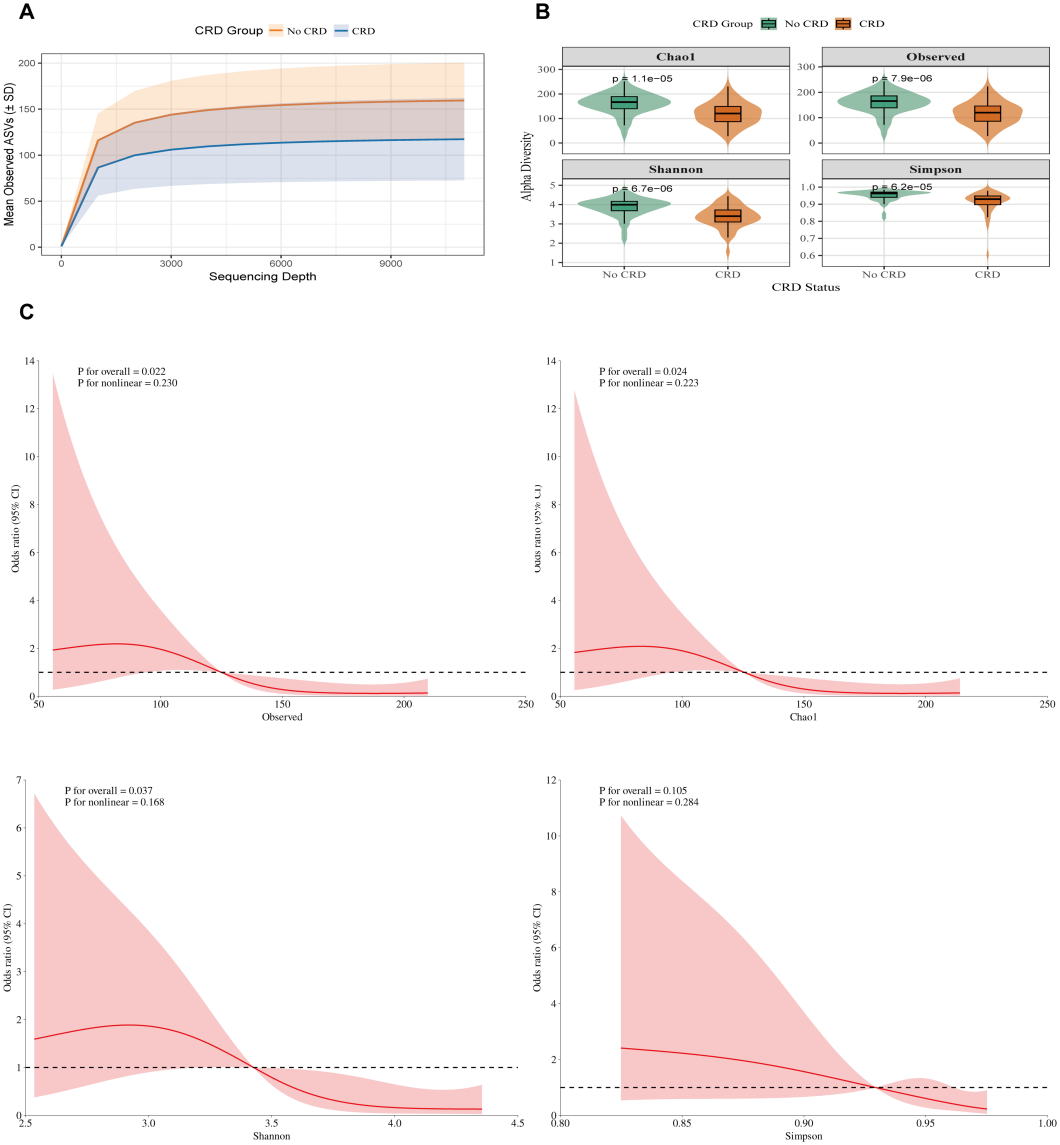
Alpha diversity between individuals with and without CRD in the hospital cohort. **(A)** Rarefaction curves showing observed ASVs by sequencing depth. **(B)** α-diversity indices (Chao1, Observed ASVs, Shannon, Simpson) comparing CRD and non-CRD groups. **(C)** Alpha diversity and CRD risk based on RCS models.

In the hospital cohort, rarefaction curves confirmed sufficient sequencing depth (Figure 4A). Alpha diversity was significantly lower in CRD patients compared with non-CRD participants, including Chao1 (*p* = 1.1 × 10^−5^), Observed species (*p* = 7.9 × 10^−6^), Shannon (*p* = 6.7 × 10^−6^), and Simpson (*p* = 6.2 × 10^−5^) indices (Figure 4B). RCS analysis, adjusted for age, smoking status, and periodontitis, indicated linear negative associations between CRD and the Observed species, Chao 1, and Shannon indices (all *p* < 0.05), while no significant association was observed for the Simpson index (*p* = 0.105) (Figure 4C).

### 3.3 Beta diversity analysis

**Figure 5:**
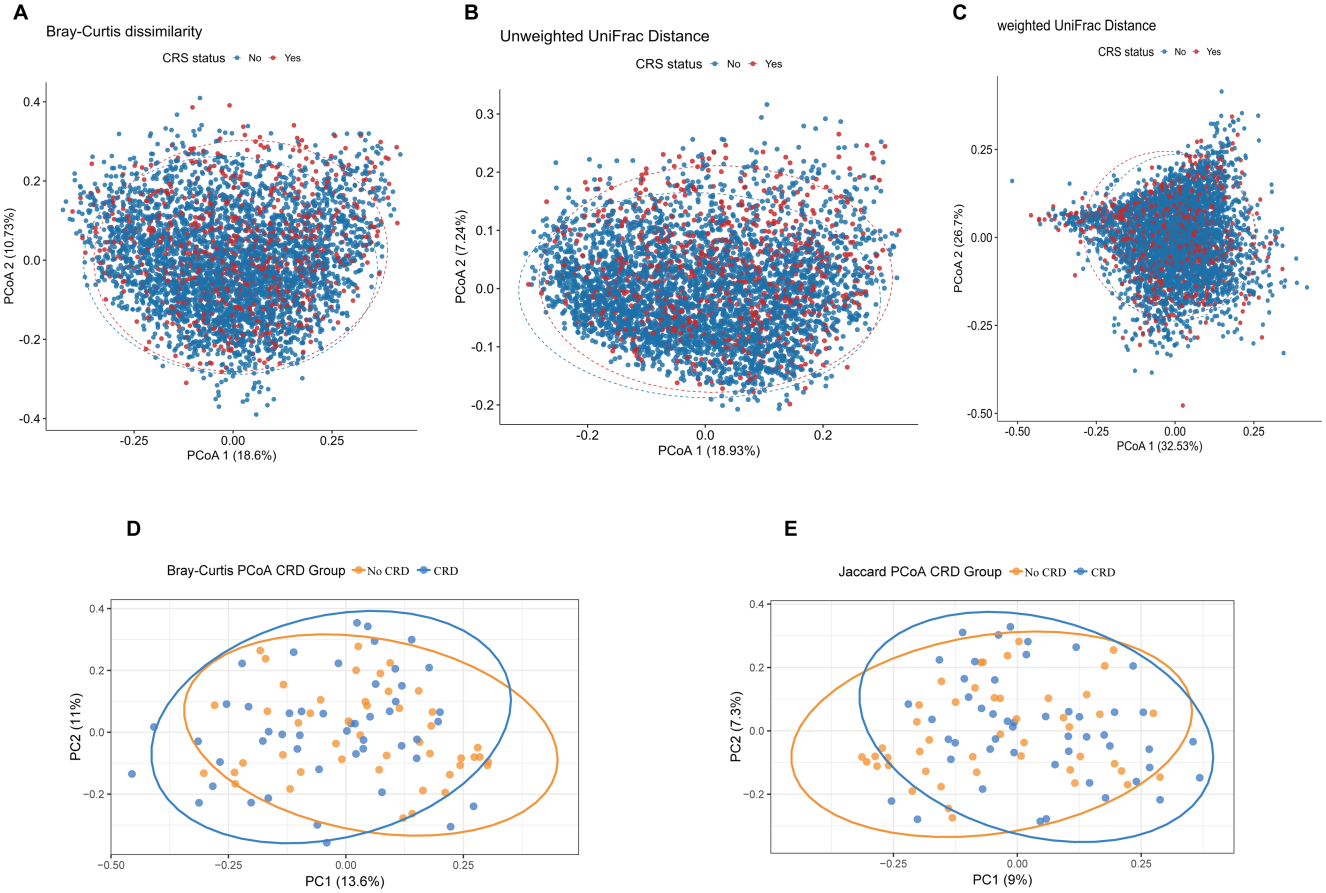
Principal coordinates analysis (PCoA) plots of beta diversity metrics comparing oral microbial community structure between CRD and non-CRD groups. **(A)** Bray–Curtis dissimilarity; **(B)** unweighted UniFrac distance; **(C)** weighted UniFrac distance. **(D)** PCoA based on Bray–Curtis distances. **(E)** PCoA based on Jaccard distances.

Beta diversity analyses were conducted to compare overall microbial community composition between participants with and without CRD in both the NHANES and hospital-based cohorts. In the NHANES cohort, principal coordinates analysis (PCoA) and PERMANOVA using Bray–Curtis dissimilarity, unweighted UniFrac, and weighted UniFrac distances showed significant differences in community structure after adjusting for demographic and lifestyle factors (Bray–Curtis: *R*^2^ = 7.95%, *p* = 0.01; Unweighted UniFrac: *R*^2^ = 5.34%, *p* = 0.01; Weighted UniFrac: *R*^2^ = 5.93%, *p* = 0.01) (Figures 5A–C). Similarly, in the hospital cohort, Bray–Curtis and Jaccard-based PCoA revealed partial separation between CRD and non-CRD participants, with PC1 and PC2 explaining 13.6 and 11.0% of variance for Bray–Curtis, and 9.0 and 7.3% for Jaccard (Figures 5D,E). PERMANOVA confirmed significant differences in microbial community structure (Bray–Curtis: *R*^2^ = 2.38%, *p* = 0.003; Jaccard: *R*^2^ = 1.98%, *p* = 0.002).

### 3.4 Genus-level differential abundance and predictive modeling of CRD in the NHANES

**Figure 6:**
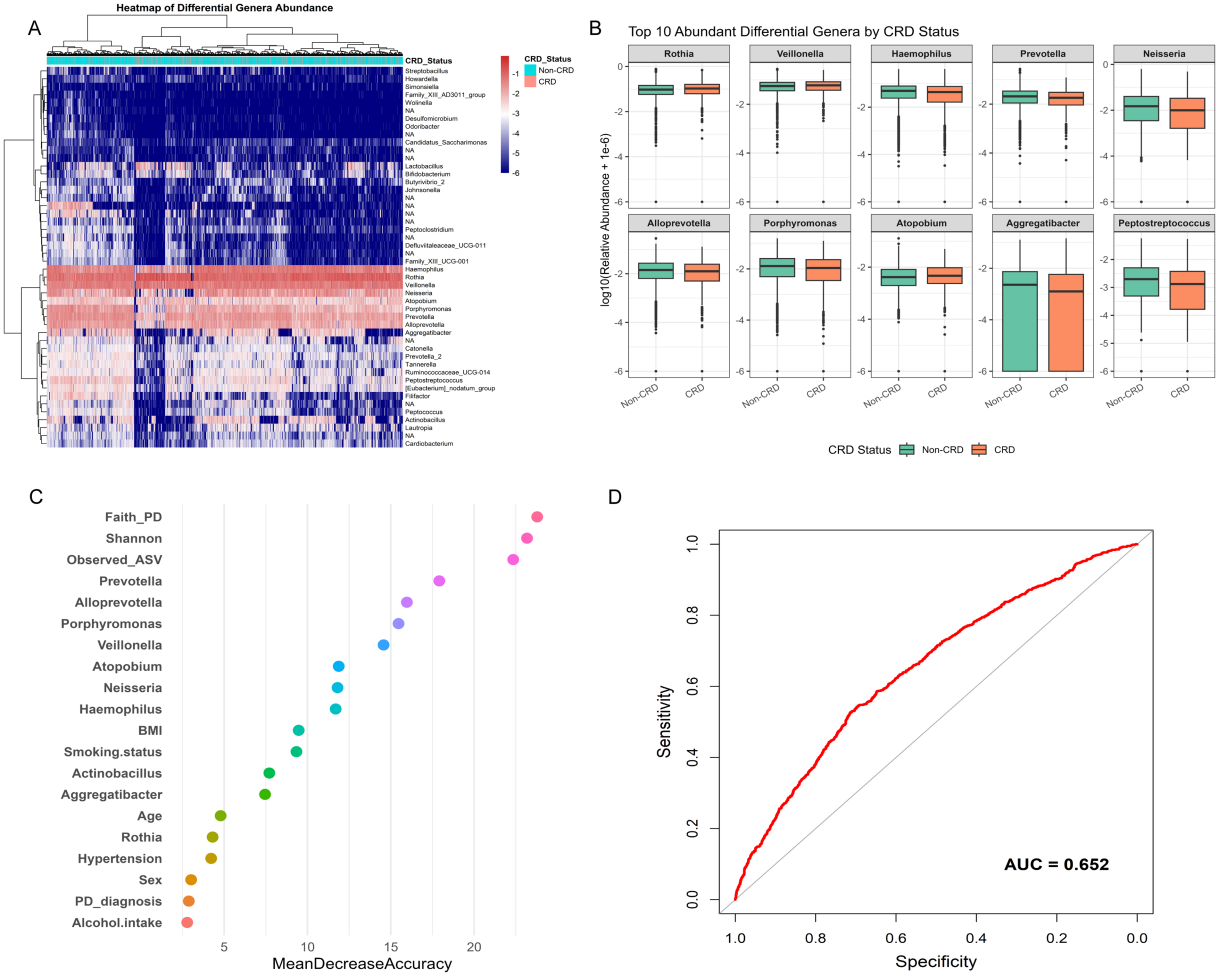
Genus-level differential abundance and predictive modeling of CRD in the NHANES. **(A)** Boxplots of top 10 differentially abundant genera between CRD and non-CRD groups. **(B)** Variable importance plot from random forest classification model. **(C)** Receiver operating characteristic (ROC) curve assessing model performance. **(D)** Receiver operating characteristic (ROC) curve assessing model performance.

To identify microbial features linked to CRD, we conducted genus-level differential abundance analysis followed by predictive modeling. After FDR correction (FDR < 0.05), 385 genera showed significant differences in relative abundance between CRD and non-CRD groups. To ensure biological relevance, we further selected genera present in at least 5% of participants, yielding 42 representative genera for hierarchical clustering. The heatmap revealed distinct microbial composition patterns between the two groups (Figure 6A). Among the top 10 differentially abundant genera, *Rothia*, *Veillonella*, and *Atopobium* were enriched in the CRD group. In contrast, *Haemophilus*, *Prevotella*, *Neisseria*, *Alloprevotella*, *Porphyromonas*, *Aggregatibacter*, and *Peptostreptococcus* were more abundant in the non-CRD group. These genera spanned major phyla such as Actinobacteria, Firmicutes, Bacteroidetes, and Proteobacteria. Boxplots clearly showed the distinct abundance patterns of these genera between groups (Figure 6B).

We then incorporated the 10 genera, two alpha diversity indices (Observed ASVs and Faith’s PD), and seven key clinical variables (age, sex, smoking status, hypertension, alcohol use, BMI, and periodontal status) into a random forest classification model. Variable importance analysis showed that both microbial genera and diversity indices played significant roles in model accuracy (Figure 6C). The receiver operating characteristic (ROC) curve of this combined model yielded an area under the curve (AUC) of 0.652, indicating moderate ability to distinguish between CRD and non-CRD participants (Figure 6D).

### 3.5 Genus-level compositional differences in the hospital cohort

**Figure 7:**
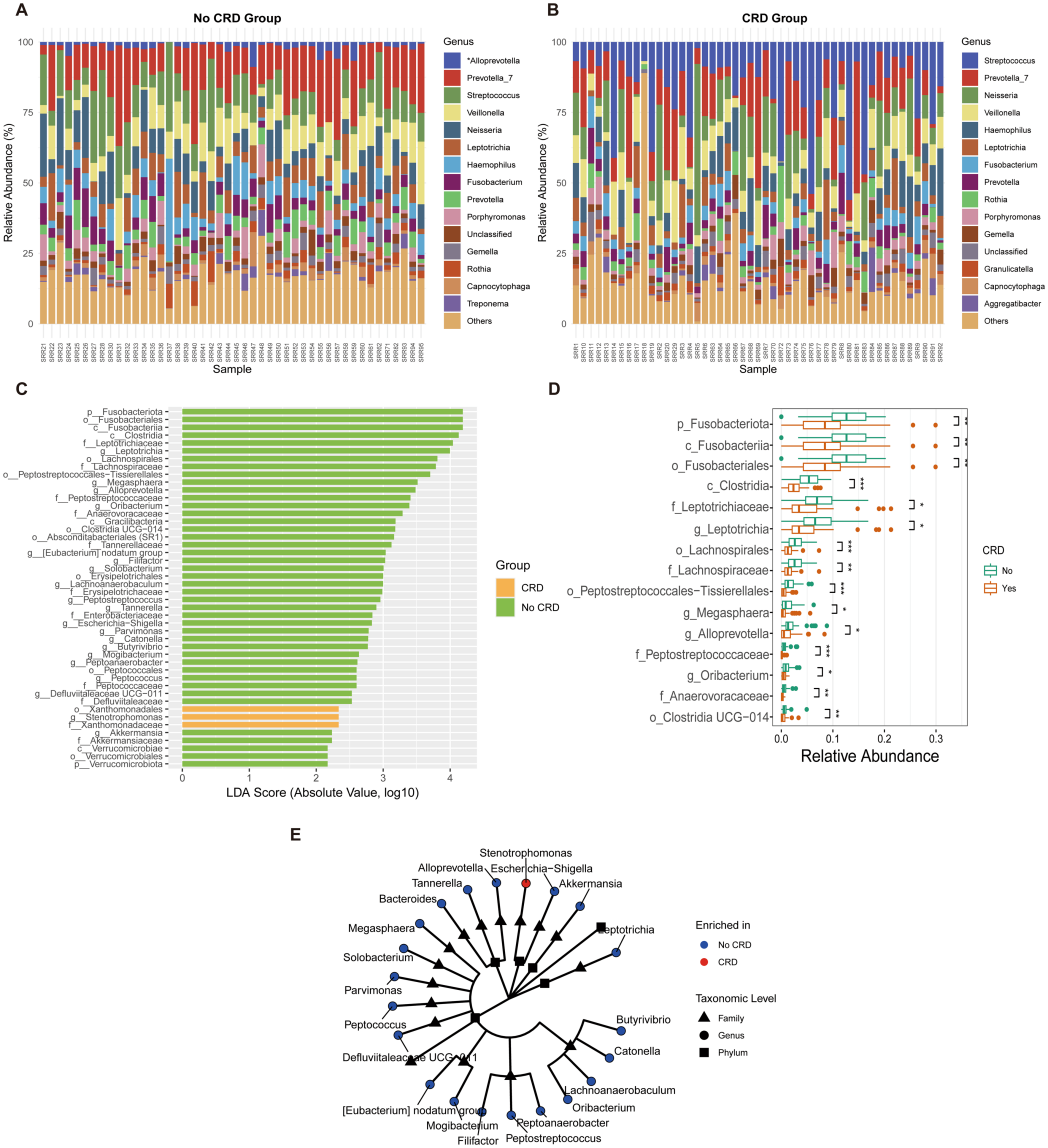
Genus-level composition and differential analysis of the oral microbiota in CRD and non-CRD groups in the hospital cohort. **(A,B)** Stacked bar plots of the relative abundance of predominant genera in non-CRD and CRD participants. **(C)** Linear discriminant analysis (LDA) scores of taxa identified by LEfSe (LDA > 2). **(D)** Boxplots showing relative abundance of representative differential genera. **(E)** Cladogram illustrating the phylogenetic relationships of taxa with significant differences between groups.

At the genus level, distinct shifts in microbial composition were observed between CRD and non-CRD groups (Figures 7A,B). In the non-CRD group, genera such as *Alloprevotella*, *Prevotella*, and *Veillonella* were more abundant, whereas the CRD group was enriched in potential pathogenic taxa including *Fusobacterium*, *Leptotrichia*, and *Porphyromonas*.

LEfSe analysis (LDA score > 2) identified taxa that discriminated between the two groups (Figure 7C). Genera such as *Alloprevotella* and *Peptostreptococcus* were enriched in the non-CRD group, while *Fusobacterium* and *Leptotrichia* were significantly associated with CRD.

Boxplot analysis confirmed significant differences in the relative abundances of the identified genera (Figure 7D). *Alloprevotella* showed higher relative abundance in the non-CRD group (*p* < 0.05), whereas *Fusobacterium* and *Leptotrichia* were markedly enriched in the CRD group (*p* < 0.01).

Phylogenetic analysis further demonstrated that taxa enriched in the non-CRD group clustered into coherent modules, including genera such as *Alloprevotella*, *Megasphaera*, *Parvimonas*, and *Peptostreptococcus* (Figure 7E). In contrast, CRD-enriched taxa, represented by *Escherichia–Shigella*, were relatively isolated within the phylogenetic tree.

### 3.6 Sensitivity analysis in the NHANES

In the NHANES cohort, sensitivity analyses excluding participants who reported recent antibiotic use, with HEI-2015 additionally included as a covariate, showed results consistent with the main analyses (Supplementary Figures S1–S4 and Supplementary Table S2).

## 4 Discussion

Across both the population-based NHANES analysis and our hospital cohort, higher oral microbial alpha diversity was consistently associated with lower odds of CRD. This association remained significant after adjusting for multiple potential confounders, and the relationship appeared linear across different diversity metrics. Beta diversity analyses further revealed clear separation between CRD and non-CRD participants, suggesting global alterations in community composition. At the genus level, specific taxa were differentially enriched in CRD versus non-CRD individuals, reflecting disease-related microbial dysbiosis. Collectively, the concordant findings from two independent cohorts strengthen the evidence for a robust association between reduced oral microbial diversity and increased CRD risk.

In the NHANES analysis, weighted logistic regression models showed that higher oral *α*-diversity was associated with lower CRD risk. Each standard deviation increase in Observed ASVs and Faith’s PD corresponded to 19 and 17% reductions in CRD risk, respectively, while the Shannon index was only weakly associated with reduced risk. These findings indicate that greater microbial richness and evenness might be linked to resilience against chronic respiratory inflammation. Importantly, the inverse associations remained robust in subgroup analyses, particularly among non-Hispanic White and Mexican American populations. This observation is consistent with prior reports of racial differences in oral microbial diversity (22–27). Regarding β-diversity, both PCoA and PERMANOVA analyses revealed significant structural differences in the oral microbiome between CRD and non-CRD individuals using Bray–Curtis, weighted UniFrac, and unweighted UniFrac distances (all *p* < 0.01), underscoring the presence of microbial dysbiosis (28–31).

At the genus level, *Rothia* and *Veillonella* were enriched in CRD cases, whereas *Haemophilus*, *Prevotella*, and *Neisseria* were more common in non-CRD participants. These distribution patterns align with prior studies showing that common oral genera, including *Veillonella*, *Prevotella*, *Fusobacterium*, and *Actinomyces*, can migrate to the lower respiratory tract and potentially influence respiratory health (24, 32, 33), which is consistent with the hypothesis that the oral cavity serves as a microbial reservoir for respiratory disease. Consistent with NHANES, the hospital cohort also showed that Fusobacterium, Leptotrichia, and Rothia were enriched in CRD patients, whereas Prevotella, Haemophilus, Neisseria, and Alloprevotella were more abundant in non-CRD individuals. These overlapping patterns reinforce the robustness of these taxa as potential microbial markers associated with CRD. However, the LEfSe analysis yielded partially different results. Only two genera in the non-CRD group—*Alloprevotella* and *Peptostreptococcus*—overlapped with the NHANES findings. This discrepancy may reflect methodological differences: NHANES emphasized abundant and clearly differentially expressed genera, whereas LEfSe integrates features across multiple taxonomic ranks (phylum, class, order, family, and genus), thereby attenuating genus-level signals.

Additionally, the random forest model showed moderate predictive performance. This likely reflects the study population, which is drawn from a general, mostly healthy cohort. In such population-based settings, differences between CRD and non-CRD individuals are subtler than in hospital cohorts or case–control studies, making prediction inherently more challenging. Despite this, the model still identifies relevant microbial and clinical features, supporting the epidemiological relevance of the findings. Moreover, predictive performance could potentially be improved in future studies by incorporating additional features, such as lifestyle factors or multi-omics data.

Nevertheless, both methods consistently highlighted *Alloprevotella* and *Peptostreptococcus* as being depleted in the CRD group across cohorts. This convergence suggests their possible involvement in respiratory health rather than a definitive protective role. The oral microbiome serves as an important reservoir of respiratory pathogens. Bacteria from dental plaque, periodontal pockets, and saliva can be aspirated into the lower respiratory tract, where they may trigger or exacerbate conditions such as aspiration pneumonia and COPD. The pathogenic mechanisms involve immune modulation, particularly the balance between Th1 and Th2 responses. Oral pathogens can stimulate airway epithelial cells to produce pro-inflammatory cytokines (e.g., TNF-α, IL-1β, and IL-6) and regulate mucus secretion (34, 35). In addition, microbial enzymes and cellular products from the oral microbiome can disrupt the respiratory mucosal barrier, facilitating pathogen colonization and increasing the risk of infection (36, 37). Emerging evidence also highlights complex interactions between commensal oral microbes and respiratory pathogens, with some species enhancing virulence and others producing inhibitory substances that limit pathogen growth (38, 39).

In this context, *Alloprevotella* may contribute to mucosal barrier maintenance and inflammation modulation through the production of short-chain fatty acids (SCFAs), such as acetate and succinate. SCFAs have also been shown to regulate Th17-mediated pathways, which are strongly implicated in COPD and asthma (40). Similarly, *Peptostreptococcus* may regulate microbial community balance and modulate host immunity via metabolic cross-feeding interactions with other commensals. By stabilizing the oral ecosystem, it may indirectly limit the expansion of pathobionts such as Rothia and Fusobacterium, which were enriched in CRD patients (41). Their reduction may reflect ecological shifts that compromise mucosal defense and promote inflammation, but causality remains uncertain, ultimately increasing susceptibility to chronic respiratory inflammation. These mechanistic explanations are speculative and should be further examined in longitudinal and experimental studies. Moreover, recent evidence suggests that the interaction between the microbiome and host vitamin D metabolism plays an important role in modulating immune responses, including autoimmunity and chronic inflammation. Vitamin D influences both innate and adaptive immunity by regulating antimicrobial peptide expression and promoting immune tolerance. Alterations in oral microbial composition could therefore affect vitamin D–mediated mucosal immunity along the oral–lung axis. Conversely, vitamin D deficiency has been associated with dysbiosis and impaired epithelial barrier function, which may exacerbate respiratory inflammation. These findings, as discussed by Murdaca et al. (42–44), highlight the complex bidirectional interplay between vitamin D signaling and the microbiome in shaping systemic and respiratory immune responses.

This study, based on a nationally representative NHANES sample and an independent hospital cohort, is the first to systematically assess the association between the oral microbiome and CRD, identifying key genera and exploring potential biological mechanisms. The use of two complementary cohorts enhances the robustness and generalizability of the findings.

However, several limitations should be noted. First, both cohorts were cross-sectional in nature, which limits causal inference and precludes assessment of temporal changes in the oral microbiome during disease progression. Second, although the NHANES sample offers broad population representativeness, it was restricted to only two cycles, while the hospital cohort—although valuable for validation—had a more limited sample size and may be subject to selection bias. Third, although mouthwash samples are widely used in oral microbiome studies and can capture overall microbial diversity (45, 46), they may not fully reflect microbial communities in specific oral niches such as subgingival or tongue dorsum areas. Finally, due to the lack of lung microbiome data, this study could not directly validate the biological pathways linking the oral and pulmonary systems (“oral–lung axis”) in CRD, and mechanistic interpretations remain largely based on prior evidence.

In addition, the moderate predictive performance of the random forest model and the reliance on LEfSe for differential abundance analysis should be acknowledged when interpreting the findings. LEfSe was chosen due to its wide use in oral microbiome studies and its suitability for validating NHANES findings in our hospital cohort, whereas methods such as DESeq2 or ANCOM may be limited by the smaller sample size. Future studies could enhance prediction by incorporating additional variables, complementary differential abundance methods, or multi-omics data.

## 5 Conclusion

In this study, using both a nationally representative NHANES sample and an independent hospital cohort, we observed a consistent association between the oral microbiome and CRD. CRD patients exhibited reduced α-diversity, distinct β-diversity patterns, and differential enrichment of specific bacterial genera. Notably, the depletion of Alloprevotella and Peptostreptococcus was consistent across cohorts, highlighting robust microbial signatures associated with CRD and supporting the relevance of the oral microbiome and the “oral–lung axis” in respiratory health.

## Data Availability

The datasets presented in this study can be found in online repositories. The names of the repository/repositories and accession number(s) can be found in the article/Supplementary material.

## Supplementary material

SUPPLEMENTARY FIGURE S1 Dose–response relationships between oral microbial alpha diversity indices and CRD risk assessed by restricted cubic spline models. **(A)** Observed ASVs; **(B)** Faith’s phylogenetic diversity; **(C)** Shannon index; **(D)** Simpson index.

SUPPLEMENTARY FIGURE S2 Subgroup analyses of associations between alpha diversity indices and CRD risk. **(A)** Observed ASVs; **(B)** Faith’s phylogenetic diversity.

SUPPLEMENTARY FIGURE S3 Principal coordinates analysis (PCoA) plots of beta diversity metrics comparing oral microbial community structure between CRD and non-CRD groups. **(A)** Bray–Curtis dissimilarity; **(B)** unweighted UniFrac distance; **(C)** weighted UniFrac distance.

SUPPLEMENTARY FIGURE S4 Genus-level differential abundance and predictive modeling of CRD. **(A)** Boxplots of top 10 differentially abundant genera between CRD and non-CRD groups. **(B)** Variable importance plot from random forest classification model. **(C)** Receiver operating characteristic (ROC) curve assessing model performance.
