# Supplementary figures and images for: Oral microbiome dysbiosis is associated with chronic respiratory diseases: evidence from a population-based study and a hospital cohort
Source: Front Public Health. 2025 Oct 30;13:1696041. doi: 10.3389/fpubh.2025.1696041 (PMC12612837; doi:10.3389/fpubh.2025.1696041)

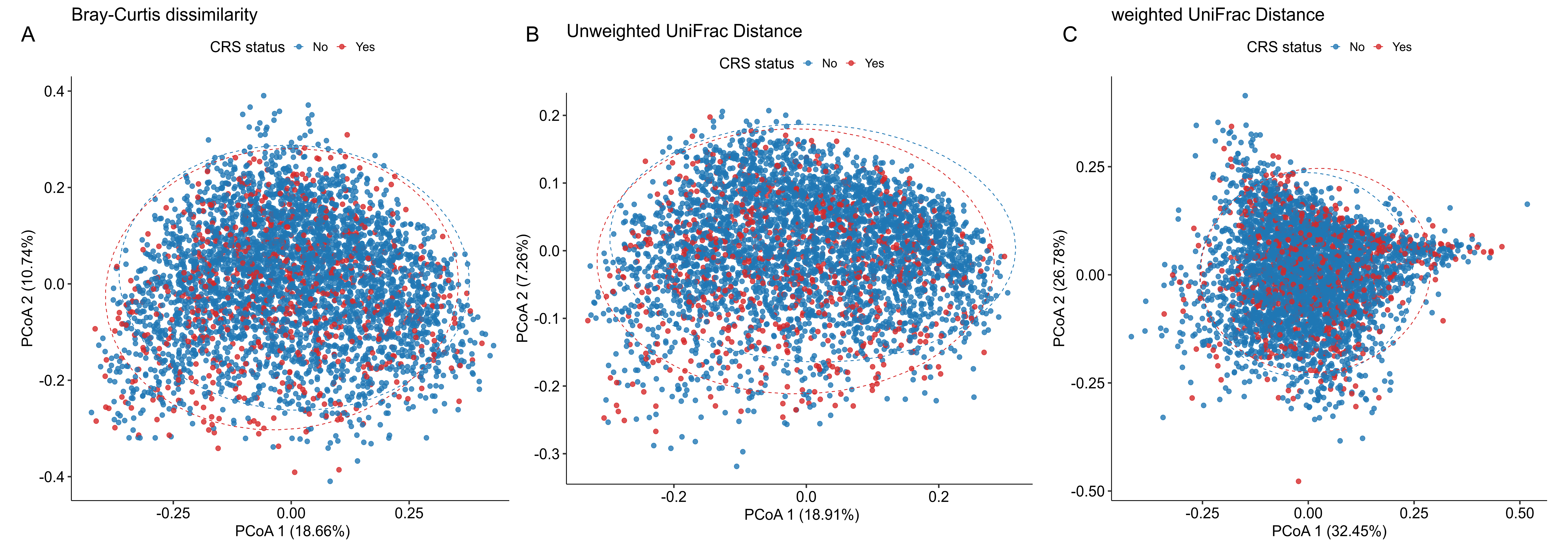

Supplement: SUPPLEMENTARY FIGURE S2 — Subgroup analyses of associations between alpha diversity indices and CRD risk. (A) Observed ASVs; (B) Faith’s phylogenetic diversity. [file Image_2.tif]

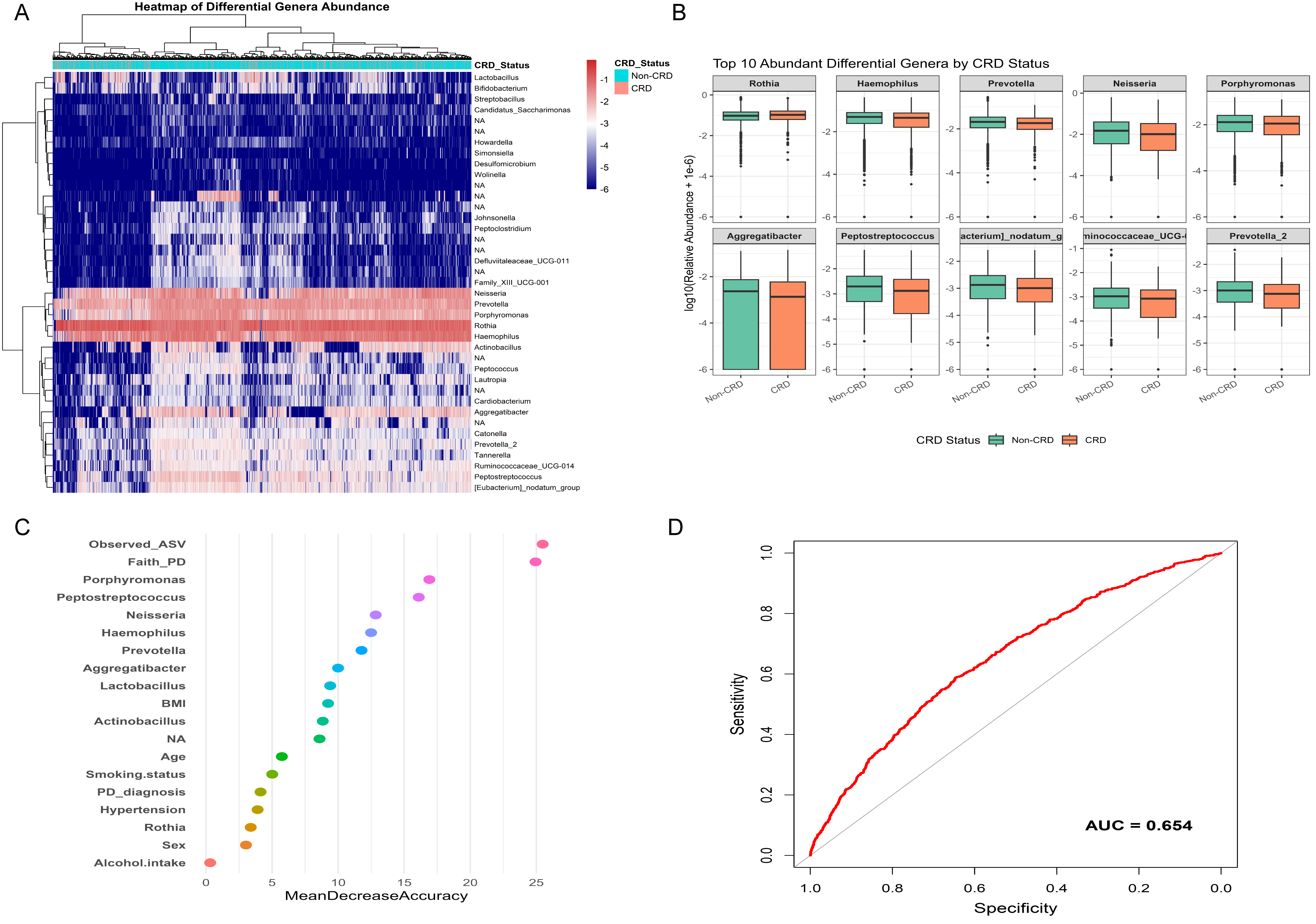

Supplement: SUPPLEMENTARY FIGURE S3 — Principal coordinates analysis (PCoA) plots of beta diversity metrics comparing oral microbial community structure between CRD and non-CRD groups. (A) Bray–Curtis dissimilarity; (B) unweighted UniFrac distance; (C) weighted UniFrac distance. [file Image_3.tif]

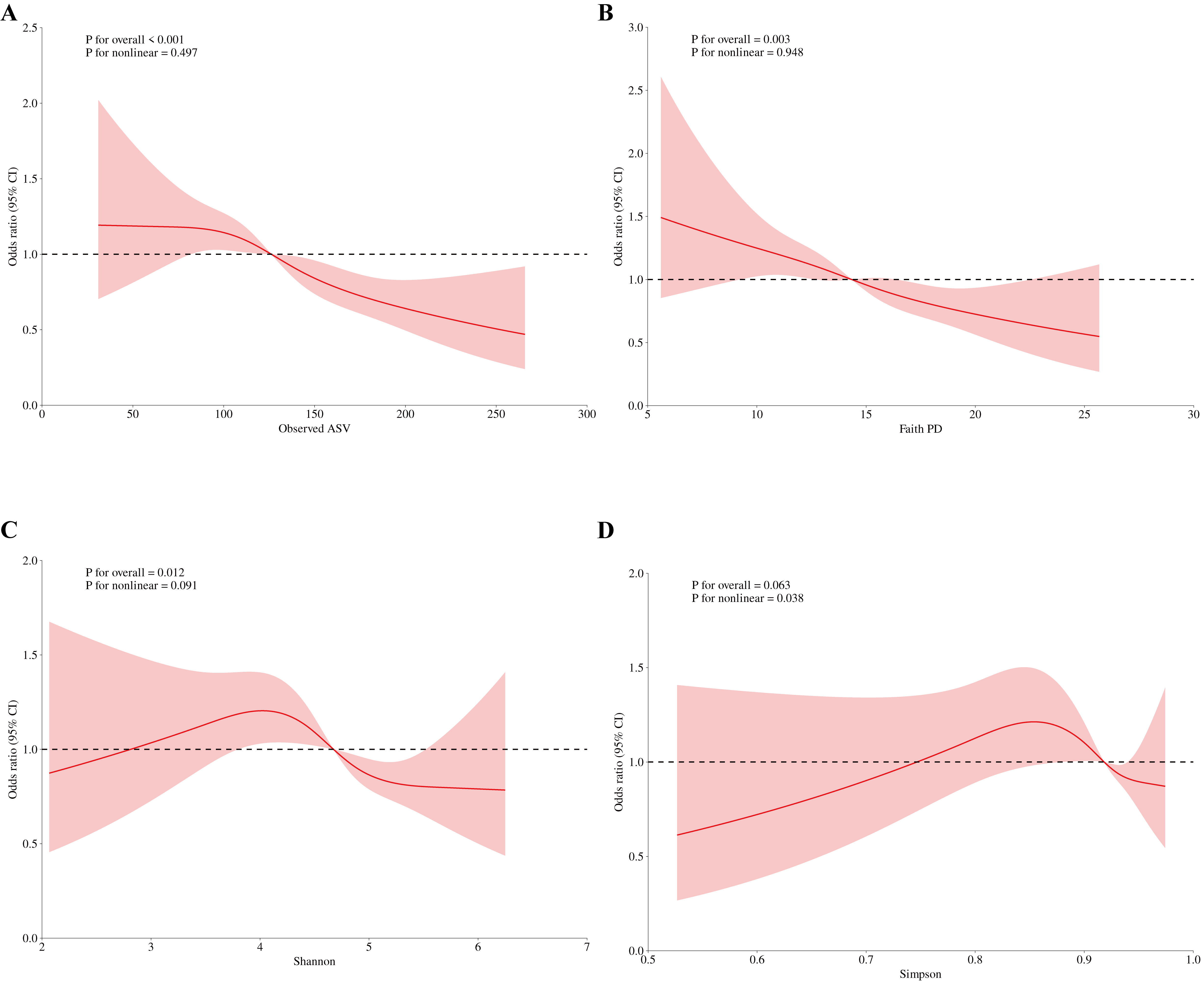

Supplement: SUPPLEMENTARY FIGURE S4 — Genus-level differential abundance and predictive modeling of CRD. (A) Boxplots of top 10 differentially abundant genera between CRD and non-CRD groups. (B) Variable importance plot from random forest classification model. (C) Receiver operating characteristic (ROC) curve assessing model performance. [file Image_4.tif]
