# Supplementary material for: Oral microbiome dysbiosis is associated with chronic respiratory diseases: evidence from a population-based study and a hospital cohort
Source: Front Public Health. 2025 Oct 30;13:1696041. doi: 10.3389/fpubh.2025.1696041 (PMC12612837; doi:10.3389/fpubh.2025.1696041)
Supplement: Supplementary file 5 [file Table_1.docx]

Table S1. Diagnostic Criteria for Hypertension, Diabetes Mellitus, and Hyperlipidemia

| Condition | Diagnostic Criteria |
| --- | --- |
| Hypertension (HT) | 1. Blood Pressure Measurement: Three or more measurements with an average value of SBP ≥ 140 mmHg or DBP ≥ 90 mmHg after 5 minutes of rest. Measurements performed by trained personnel according to standardized protocols. 2. Medication/History Criteria: Any of the following conditions even if current blood pressure is below 140/90 mmHg: on antihypertensive medication, previously diagnosed with hypertension by a physician, self-reported use of hypertension prescription drugs. 3. Diagnostic Process: Confirmed by multiple measurements on different days to avoid misdiagnosis from occasional blood pressure spikes. |
| Diabetes Mellitus (DM) | 1. Clinical Diagnosis: Diagnosed by a doctor as Diabetes Mellitus (DM). 2. Laboratory Indicators: HbA1c ≥ 6.5%; Fasting Blood Glucose ≥ 7.0 mmol/L; Random Blood Glucose ≥ 11.1 mmol/L; 2-hour OGTT ≥ 11.1 mmol/L. 3. Treatment: Currently on diabetes medication or insulin therapy. |
| Hyperlipidemia | 1. Laboratory Criteria: Diagnosed based on the following blood lipid levels: Total Cholesterol (TC) ≥ 200 mg/dL (5.2 mmol/L), LDL-C ≥ 130 mg/dL (3.4 mmol/L), HDL-C (Males <40 mg/dL, Females <50 mg/dL), TG ≥ 150 mg/dL (1.7 mmol/L). 2. Medication/History Criteria: Diagnosed even if current blood lipid levels are normal, if any of the following: on lipid-lowering medication (e.g., statins, fibrates, ezetimibe), previously diagnosed with hyperlipidemia or high cholesterol by a physician. |
