# Supplementary material for: Oral microbiome dysbiosis is associated with chronic respiratory diseases: evidence from a population-based study and a hospital cohort
Source: Front Public Health. 2025 Oct 30;13:1696041. doi: 10.3389/fpubh.2025.1696041 (PMC12612837; doi:10.3389/fpubh.2025.1696041)
Supplement: Supplementary file 6 [file Table_2.docx]

| **CRD** | **Model 1** | |  | **Model 2** | |  | **Model 3** | |
| --- | --- | --- | --- | --- | --- | --- | --- | --- |
|  | **OR (95% CI**) | ***p*-value** |  | **OR (95% CI**) | ***p*-value** |  | **OR (95% CI**) | ***p*-value** |
| Observed ASVs |  |  |  |  |  |  |  |  |
| Per SD increase | 0.82 (0.75, 0.91) | <0.001 |  | 0.86 (0.77, 0.96) | 0.009 |  | 0.83 (0.71, 0.97) | 0.025 |
| Q1 | Ref |  |  | Ref |  |  | Ref |  |
| Q2 | 0.79 (0.61, 1.04) | 0.088 |  | 0.83 (0.63,1.10) | 0.181 |  | 0.84 (0.59, 1.20) | 0.260 |
| Q3 | 0.67 (0.52, 0.87) | 0.004 |  | 0.73 (0.55, 0.96) | 0.028 |  | 0.68 (0.49, 0.96) | 0.032 |
| Faith’s phylogenetic diversity |  |  |  |  |  |  |  |  |
| Per SD increase | 0.84 (0.76, 0.94) | 0.002 |  | 0.87 (0.78, 0.98) | 0.028 |  | 0.84 (0.71, 0.99) | 0.041 |
| Q1 | Ref |  |  | Ref |  |  | Ref |  |
| Q2 | 0.78 (0.59, 1.05) | 0.096 |  | 0.80 (0.59, 1.08) | 0.129 |  | 0.76 (0.52, 1.13) | 0.141 |
| Q3 | 0.69 (0.54, 0.88) | 0.004 |  | 0.74 (0.56, 0.98) | 0.029 |  | 0.67 (0.48, 0.95) | 0.030 |
| Shannon-Weiner index |  |  |  |  |  |  |  |  |
| Per SD increase | 0.90 (0.81, 1.01) | 0.063 |  | 0.92 (0.82, 1.04) | 0.166 |  | 0.91 (0.78, 1.06) | 0.177 |
| Q1 | Ref |  |  | Ref |  |  | Ref |  |
| Q2 | 0.93 (0.67, 1.28) | 0.627 |  | 0.96 (0.68, 1.35) | 0.816 |  | 0.99 (0.67, 1.50) | 0.991 |
| Q3 | 0.78 (0.57, 1.07) | 0.118 |  | 0.82 (0.59, 1.15) | 0.243 |  | 0.81 (0.54, 1.21) | 0.248 |
| Simpson index |  |  |  |  |  |  |  |  |
| Per SD increase | 0.99 (0.89, 1.10) | 0.776 |  | 0.99 (0.89, 1.10) | 0.812 |  | 0.97 (0.86, 1.11) | 0.622 |
| Q1 | Ref |  |  | Ref |  |  | Ref |  |
| Q2 | 1.01 (0.76, 1.33) | 0.960 |  | 1.02 (0.75, 1.38) | 0.907 |  | 1.00 (0.70, 1.42) | 0.999 |
| Q3 | 0.80 (0.58, 1.12) | 0.181 |  | 0.80 (0.59, 1.10) | 0.181 |  | 0.79 (0.52, 1.18) | 0.195 |

Table S2. Associations between oral microbial alpha diversity indices and CRD in unadjusted and fully adjusted logistic regression models

Model 1 is unadjusted.

Model 2 is adjusted for age, sex, race/ethnicity, marital status, PIR, education and BMI.

Model 3 is further adjusted for smoking status, physical activity, alcohol intake, Flossing behavior, Mouthwash behavior, Hypertension, DM, Hyperlipidemia Periodontitis and HEI-2015.

Results are presented as odds ratios (ORs) with 95% confidence intervals (CIs).
